# Identifying Language Disorder Within a Migration Context: Development and Performance of a Pre-school Screening Tool for Children With German as a Second Language

**DOI:** 10.3389/fped.2022.814415

**Published:** 2022-03-08

**Authors:** Daniel Holzinger, Christoph Weber, Magdalena Jezek

**Affiliations:** ^1^Institute of Neurology of Senses and Language, Hospital of St. John of God, Linz, Austria; ^2^Research Institute for Developmental Medicine, Johannes Kepler University, Linz, Austria; ^3^Institute of Linguistics, University of Graz, Graz, Austria; ^4^Department for Inclusive Education, University of Education Upper Austria, Linz, Austria

**Keywords:** language screening, multilingual, migration, pre-school, German as a second language

## Abstract

**Background:**

There is a lack of accurate and practicable instruments for identifying language disorders in multilingual children in pre-school settings.

**Objective:**

To develop a language screening instrument for pre-school children who are growing up with German as their second language.

**Design:**

After the development and initial validation of a language screening tool, the new instrument (LOGiK-S) was administered to three cohorts of children (2014, 2015, 2017) with a non-German first language attending a variety of public pre-schools in Upper Austria. The screening instrument measures expressive and receptive grammatical skills in German. The final validation study included the results for 270 children for the screening measure and reference tests. A combination of a standardized comprehensive language test of grammatical skills developed for children acquiring German as a second language and a test of expressive vocabulary with the use of specific cutoffs for second language learners was applied as the gold standard for identifying language disorders.

**Results:**

The LOGiK-S screening of expressive grammar demonstrated excellent accuracy (AUC.953). The screening subscale of receptive grammar did not improve the prediction of language disorders. Using an optimized cutoff yielded a fail rate of 17%, excellent sensitivity (0.940), and specificity (0.936). Time economy and acceptance of the screening by children and screeners were mostly rated as high.

**Conclusion:**

The LOGiK-S language screening instrument assessing expressive German grammar development using bilingual norms is a valid and feasible instrument for the identification of language disorders in second language learners of German at the pre-school age.

## Introduction

With prevalence rates of about 10%, language disorders (LDs) can be considered the most frequent developmental problem in children under the age of 7 (1–4). Prevalence estimations vary because of the lack of a generally accepted definition of LD. The term developmental language disorder (DLD) was endorsed in a consensus document by Bishop et al. (5) as referring to language difficulties characterized by a lack of known biomedical etiology, functional impairment, and poor prognosis. Therefore, LD remains a diagnosis to be made by experienced clinicians able to assess different dimensions of language, the degree of impairment caused by the language difficulties, and the probability of persistence. A population study on LD in England by Norbury et al. (6) resulted in a prevalence of DLD of 7.58%. In addition, 2.34% of LDs were associated with an intellectual disability or a medical diagnosis, adding up to about 10% of LDs in total. The authors classified a child as language disordered when language performance was at least 1.5 standard deviations below the norm on at least two of five language domains. Other researchers (1, 4, 7) defined a specific LD by scores of at least −1.25 standard deviations in at least two language domains. Problems often associated with pre-school LD include increased rates of behavioral, social, and emotional difficulties (8, 9), poor academic outcomes (10), and higher risk of unemployment (11).

The prevalence of LD is expected to be the same in children growing up monolingually or multi-lingually. Multilingual children growing up in an environment with a sufficient quantity and quality of language input are no more likely to develop LD than their monolingual peers (12).

Previous research has highlighted the effectiveness of early parent-facilitated and child-directed language intervention (13–16). As a consequence, suitable and practical screening instruments are needed for the early identification of language difficulties. As the population of young children growing up bilingually grows in Europe increases, there is a pressing need for reliable measures identifying what is typical or not in their language development.

In Upper Austria, the context of this study, the proportion of children with a first language (L1) other than German has been increasing continuously in recent years. For example, the share a of children from non-German speaking countries increased in primary education within 5 years from 16% (2012–2013) to 20% (2017–2018). In 2018–2019, one out of four children attending a pre-school had a L1 other than German. Notably, this figure is much higher in urbanized areas (17). In Upper Austria, first languages are predominantly. Bosnian/Croatian/Serbian (30%) and Turkish (20%). The remaining languages include languages such as Romanian or Arabic. In Austria, public pre-schools, with German being almost exclusively the only language of instruction, can be attended from the age of 3 up to the age of 6.

There are a number of challenges involved in the development and validation of language screening tools for children who grow up in a bilingual context (18). First, the group of bilingual children is extremely heterogeneous in relation to the length of exposure to the second language, quality and quantity of input in both languages in their families and institutional settings (e.g., pre-schools), or the family's socioeconomic status and parental education level. Second, in many cases, no instruments are available for assessing children's linguistic skills in their first language (19). When instruments are available, the examiners are faced with the problem of being unfamiliar with the diversity of first languages of the children to be screened. Third, tests developed for a particular language targeting monolingual children do not apply equally to bilingual children using this language as their L1 outside their home country. In a migration context language is in a state of constant change due to contact phenomena and does not necessarily overlap in all linguistic aspects with “the same” language in a non-migration context (20, 21). In addition, L1 attrition phenomena have been described in situations with early acquisition of an L2 and a literacy acquisition restricted to the second language (22, 23). Fourth, different profiles of language difficulties in children with LD (e.g., morpho-syntactic, semantic, phonological) complicate the time-efficient and reliable identification of increased risk of LD (24).

The systematic review by Sim et al. (9) compared pre-school screening tools. It concluded that language screening instruments could improve the rate of early identification of developmental language difficulties if incorporated into routine child-health surveillance. Therefore, a nationwide language screening program including specific instruments for pre-school children growing up bilingually is essential, especially as high percentages of children with developmental difficulties are not being detected prior to school entry (9).

As a consequence of the complexity of language screening in a multilingual context, a variety of approaches have been explored. Although generally claimed, the assessment of the L1 is usually not feasible. Another option is the use of instruments to assess the acquisition of the majority language by use of bilingual norms with specific cutoffs (25). For the acquisition of German as a second language, the LiSe-DaZ [Linguistische Sprachstandserhebung Deutsch als Zweitsprache (26)] is the only available standardized language test that provides specific norms for German as an L2 taking the length of German language exposure into account. However, the LiSe-DaZ is a comprehensive language assessment rather than an instrument that can be applied for universal screening. Finally, tools constructed according to linguistic principles that can be applied across individual languages (e.g., non-word and sentence repetition) have been proposed and shown to be useful for the identification of children with increased risk of LD in bilingual contexts (27).

The aim of the present research was to develop and evaluate a screening instrument for the identification of LD in pre-school children learning German as their second language in terms of screening accuracy and feasibility within a community pre-school setting in Austria. The new screening tool assesses the acquisition of German grammar. We report the results of two studies. Study 1 was a pilot study focusing on the screening development and initial validation of the screening instrument. The aim of Study 2 was the final validation of the screening instrument by the additional use of a comprehensive reference test developed for learners of German as an L2.

## Study 1 (Pilot Study)

### Methods

#### Participants

In 2012, all children growing up with a language other than German attending 1 of 13 public pre-schools well-distributed over the central and less urbanized areas of Upper Austria were invited to participate in the pilot study (Study 1). After the exclusion of children with German as their dominant language and those with a length of German language exposure below 1 year [following (28, 29)], the final sample consisted of 112 children (49.1% girls) with a mean age of 57.4 months (SD = 4 months) and a mean length of exposure to German of 18.9 months (SD = 5.7 months). Note that the length of exposure is limited as children can be enrolled in pre-school at the earliest at the age of 3 years and the study focuses on children in their penultimate pre-school year. The most frequent first languages spoken by the participants were Bosnian/Croatian/Serbian (29.5%), Turkish (15.2%), Albanian (9.8%), Czech (7.1%), Arabic (7.1%), and Romanian (6.3%).

#### Procedures

The screening procedures were carried out by clinical linguists from the Institute of Neurology of Senses and Language and by trained students of speech-language therapy from the University for Health Professions (Fachhochschule für Gesundheitsberufe) in Linz. Before the direct screening of a child, the examiners completed a structured interview with the parents on sociodemographic factors, language use in the family, the child's dominant language(s), time of exposure to German, and pre-school attendance. After the language screening, the results were reported to the parents and the pre-school teachers. Within a maximum of 90 days, the children were tested again using standardized reference tests. The tests were administered by language experts from the Institute of Neurology of Senses and Language who were blinded to the screening results.

##### Screening Measures

As LD in German, whether acquired as first or second language, manifests itself at pre-school age particularly in morphosyntax, such as subject-verb-agreement (30), verbal inflection (31), and elimination of function words (19), LOGiK-S was used to assess the following grammatical dimensions and structures:

- *Expressive grammar* (EG) was assessed by sentence completion supported by illustrations and included verb position, verb inflection, subordinate clauses, perfect forms, determiners, comparatives, noun plurals, prepositions, questions (open and closed, wh-questions), and passive structures.
- *Receptive grammar* (RG) includes the comprehension of morpho-syntactic structures, such as intransitive clauses, prepositional phrases, coordination, pronouns, and embedded and subordinate clauses. Comprehension of the grammatical structures was assessed by having the children point at the appropriate illustration from a selection of four.

In the pilot study, the screening of RG included 20 items, and the EG subscales comprised 27 items. After exclusion of items with very low and high difficulty and low items-scale correlations, and considering the input of a group of screeners involved in the pilot study, a set of 10 items for the RG subscale and a set of 17 items for the EG subscale were used. The EG subscale showed good reliability (Cronbach's α = 0.82). In constrast, the reliability of the RG subscale was relatively poor (Cronbach's α = 0.61).

##### Reference Tests

Following other studies on LDs (1, 4, 7), a child was classified as having an LD when performance in the second language was below −1.25 SDs in at least two language domains, applying bilingual norms, and when the experienced clinicians performing the diagnosis had identified serious indications of LD in the L1 from parent interviews. This goldstandard used was the best available at the time of planning the study. We used three standardized tests to assess EG, RG, and expressive vocabulary.

1. EG skills were assessed by the plural and case marking subtests of the PDSS [(32) Patholinguistische Diagnostik bei Sprachentwicklungsstörungen] as well as the subtests for comparatives and perfect tense of the ETS 4-8 [(33); Entwicklungstest Sprache für Kinder von 4 bis 8 Jahren]. The manuals only provides *t*-values for monolingual German-speaking children. However, relying on these *t*-values would have resulted in high rates of children with atypical results (*t*-values ≤ 37.5) for the four subscales (between 50 and 70%). Therefore, we used principal component analysis (PCA) to extract a composite score based on all the subscales. The PCA yielded one component with an eigenvalue of 3.2 (80% explained variance). The loadings ranged from 0.88 to 0.92. The internal consistency (Cronbach's α) was high at 0.90. We saved the component score (i.e., z-score with M = 0 and SD = 1). Children were classified as atypical in EG if they scored in the bottom 10% (1.25 SDs) of the component score.
2. The TROG-D [German version of the Test the Reception of Grammar (34)] assesses the understanding of German grammar. Similar to the PDSS and ETS 4–8, the TROG-D only provides norm values for German-speaking monolingual children. Applying these norms to German language learners would again result in high rates (55%) of children with atypical results (*t* ≤ 37.5). Therefore, we again used the sample percentiles to identify the bottom 10% of the TROG-D scores.
3. The AWST-R [Revised Active Vocabulary Test for 3–5 year-old children, Aktiver Wortschatztest für 3- bis 5-Jährige, Revision (35)] is a standardized picture-naming test for the age range from 3.0 to 5.5 years. The items are ordered by increasing difficulty. To reduce the length of the assessment, we only used the first of the two picture folders (35 items) for the assessment of expressive vocabulary. As the AWST-R again lacks norm values for the reduced version of 35 items, we again estimated norm values based on the study data. However, because the AWST-R was applied in Study 1 and Study 2, we used pooled data from both studies to estimate norm values. The samples were pooled to achieve a larger (*n* = 400) and more representative database for calculating norm values. In short, we applied a continuous norming approach using three age groups (48–50, 51–56, and 57–62 months). Continuous norming was conducted using the Cnormj package (36) in jamovi 1.6 (37).

A teacher questionnaire was used to collect child sociodemographic information, length of pre-school attendance and the teacher's assessment of the children's German language level as compared to their peers learning German as a second language.

Following our definition of atypical scores ( ≤ 1.25 SD), in at least two of the reference tests, 11 children (9.8%) were classified as LD in the pilot study. Notably, pre-school teachers estimated the language development of eight children classified as LD to be significantly worse than that of their peers (2 children's language development was estimated as slightly worse; χ^2^_(2)_ = 18.480, *p* < 0.001, Cramers V = 0.412). The LiSe-DaZ [Linguistic Language Assessment—German as a Second Language (26)] used as reference test in Study 2 was not available when Study 1 was planned.

#### Statistical Analyses

First, we reported descriptive statistics for the subscales. In a second step, we applied receiver operator characteristic (ROC) analyses to evaluate the diagnostic accuracy of the subscales. Following Swets (38), AUCs ≥ 0.9 are regarded as excellent, AUCs ≥ 0.8 and <0.9 as good, AUCs ≥ 0.7 and <0.8 as fair, and tests with AUCs <0.7 as poor. We used the bootstrapped test for paired ROC curves—as implemented in the pROC package (39) in R—to compare the AUCs between the subtests. In the next step, logistic regression was applied to investigate whether both subscales independently contribute to the prediction of LD. Finally, we determined an optimal cutoff score using the R-OptimalCutpoints package (40) and estimated the following diagnostic accuracy statistics: sensitivity (Se), specificity (Sp), positive predictive values (PPV), negative predictive values (NPV), and diagnostic likelihood ratios for positive and negative screening results (DLR+ and DLR–, respectively). Following Plante and Vance (41), Se and Sp ≥ 0.90 indicate good diagnostic accuracy, and Se and Sp ≥ 0.80 are regarded as fair. Values below 0.80 indicate an unacceptably high rate of misclassification. DLR+ and DLR– are alternative measures of diagnostic accuracy and have the advantage that—unlike predictive values—they do not depend on the prevalence of the disorder under investigation (42) DLR+ indicates the multiplicative change in the pre-screening odds of having an LD given a positive screening result (i.e., post-screening odds = DLR+ × pre-screening odds) and DLR– is the change in the pre-screening odds of having an LD given a negative screening result (post-screening odds = DLR– × pre-screening odds). DLR+ values ≥ 10 and DLR– ≤ 0.1 indicate large changes in pre-screening odds, DLR+ ≤ 10 and > 5, and DLR– > 0.1 and ≤ 0.2 indicate moderate changes, DLR+ ≤ 5 and >2, and DLR– > 0.2 and ≤ 0.5 indicate small changes. DLR+ <2 and DLR– > 0.5 are rarely important (43). The logistic regression and descriptive analyses were conducted using Jamovi 1.6 (37).

The whole study project (Study 1 and Study 2) was approved by the hospital's ethics commission “Ethikkommission Barmherzige Schwestern und Barmherzige Brüder.” All parents gave their written consent to their children's participation in the study.

### Results

#### Descriptive Statistics

**Figure 1:**
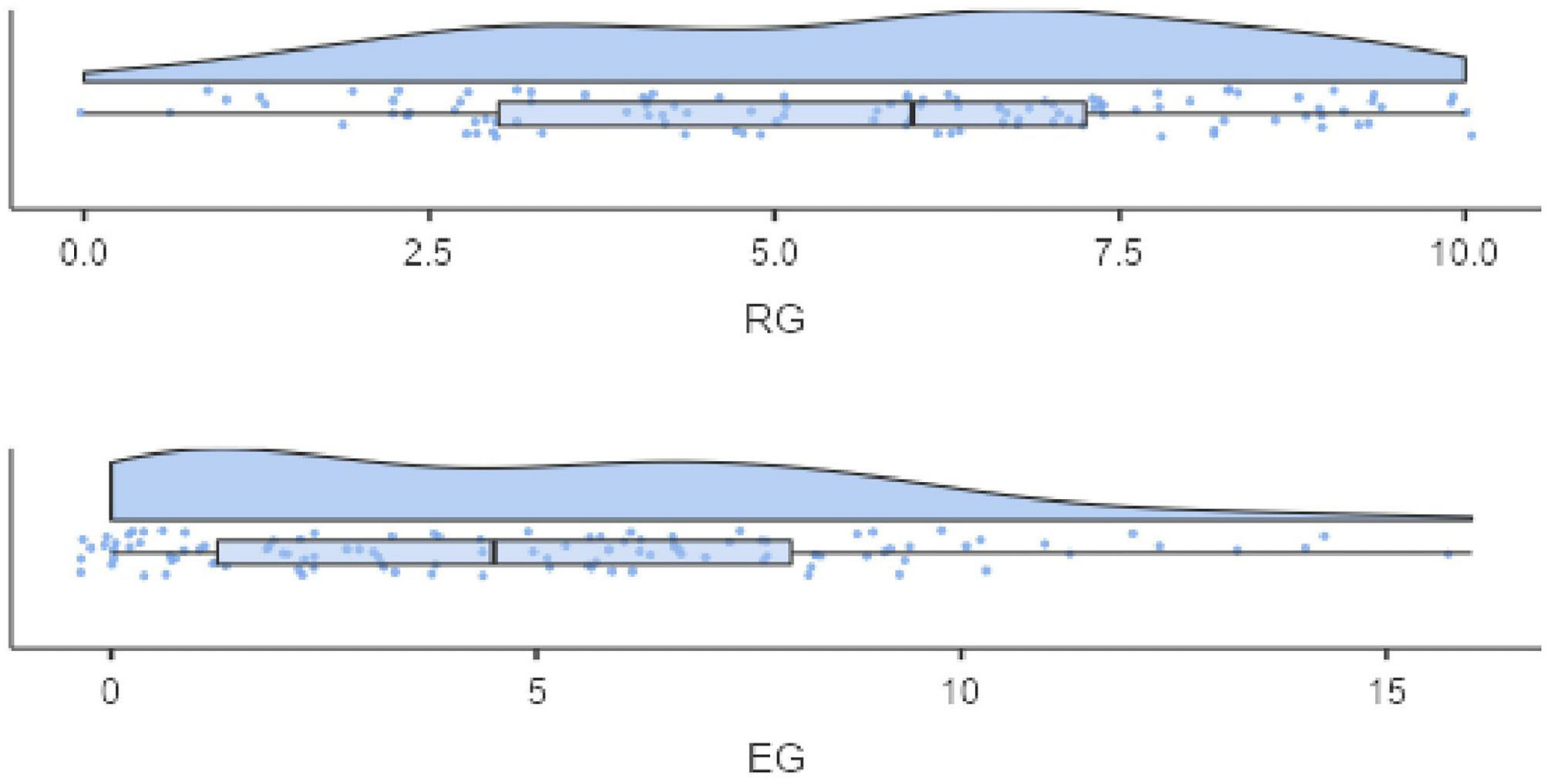
Distribution of the screening subscales—Study 1.

The distribution of the screening subscales is depicted in Figure 1. The mean of the RG subscale (M = 5.60, SD = 2.52) is above the theoretical mean of 5, indicating the relative ease of the receptive grammar items. In contrast, the mean of the EG subscale (M = 4.85, SD = 3.84) is clearly below the theoretical scale mean of 7.5, indicating that the items of the EG subscale are more difficult. Moreover, the distribution of the EG subscale appears left-censored, indicating that children with a very low EG proficiency all score at the minimum of the EG scale. The correlations of screening variables and reference tests are provided as supplement.

#### Reliabilty

The EG subscale showed good internal consistency (Cronbach's α = 0.82). In contrast, the internal consistency of the RG subscale was relatively poor (Cronbach's α = 0.61).

#### Criterion Validity

Both subscales moderately correlate with LD. The point-biserial correlation (r_pb_) is −0.317 (*p* < 0.001) for RG and −0.384 (*p* < 0.001) for EG. The AUC is fair for RG [0.793, DeLong 95% confidence interval (CI) = (0.623, 0.786)] and excellent for EG [0.912, DeLong 95% CI = (0.857–0.967)]. However, a bootstrapped test for paired ROC curves shows that the AUCs for EG and RG do not differ significantly (D = −1.401, *p* > 0.05). Next, we applied logistic regression to evaluate the independent contribution of RG and EG to LD. Results reveal a significant effect of EG only [b = −1.130, *p* < 0.05; OR = 0.323; 95% CI = (0.136, 0.770)], whereas the effect for RG was not significant [b = −0.164, *p* > 0.05; OR = 0.849; 95% CI = (0.595, 1.212)]. Thus, RG was not found to contribute independently to the prediction of LD.

#### Cutoff Estimation

Subsequently, we focused on the selection of suitable cutoff values. Due to the non-significant contribution of RG to the prediction of LD, we focused only on EG. Using the “SpEqualSe” criterion (i.e., specificity equals sensitivity) in the Optimal Cutoff Package (40), a cutoff value of 1 turned out to be the most efficient. This cutoff results in acceptable diagnostic accuracy statistics. Sensitivity was high at 0.910 [95% CI = (0.587, 0.998)], specificity was 0.818 [95% CI = (0.728, 0.889)], PPV was 0.357 [95% CI = (0.248, 0.960)], NPV was 0.988 [95% CI = (0.920, 0.993)], DLR+ was 5.000 [95% CI = (3.164, 7.903)], and DLR- was 0.111 [95% CI = (0.017, 0.722)]. Other cutoff values seemed inappropriate because a cutoff of 2 would have resulted in a sensitivity of 1, but a low specificity of 0.707, and a cutoff of 0 would have yielded a low sensitivity of 0.636.

## Study 2 (Validation Study)

### Methods

#### Participants

A total of 443 children in their penultimate year of pre-school were recruited, with parental consent, from 27 public pre-schools in the central area of Upper Austria. For practical reasons, the selected pre-schools were mostly located in the urban central area of Upper Austria, which is characterized by a high proportion of non-German-speaking children (17). Data were collected over a period of 3 years due to limited human resources in the research team and to avoid overburdening the collaborating pre-schools. Participation was voluntary at the pre-school level, and there was no selection of the children. Speech and language therapists from Upper Austria responsible for language screenings in the pre-schools were trained to administer the new measure. They performed the screening in three different test periods (Sample A: 2014, Sample B: 2015, and Sample C: 2017), but did not differ in terms of recruitment (except for the 2017 cohort, which included only children from pre-schools located in the city of Linz). According to parent reports, all the included children had a dominant first language other than German and were therefore acquiring German as a second language (L2). As the new screening tool was intended to identify children with any LD (specific and non-specific) children with additional developmental difficulties (such as hearing loss, cognitive delay, autism-spectrum-disorder) were included in the study sample. Fifty children were excluded because they had <12 months of institutionalized exposure to German. Another 73 children were excluded because of missing data on length of exposure. In addition, 50 children were excluded due to incomplete data for screening or reference tests. Time of exposure was operationalized as the institutionalized contact time (i.e., number of months children were attending pre-schools) because most children are first significantly exposed to German when they enter pre-school. In addition, it was not possible to obtain reliable parent information, and the inclusion of valid parent information on language exposure in the study [e.g., using parent diaries or interviews (44, 45)] was not considered feasible for developing a measure intended for universal screening.

Finally, 270 children were included in Study 2 (mean age = 58.5 months, SD = 3.67; 50% females) (Table 1). The children had on average 20.9 months (SD = 6.65) of institutionalized exposure to German. The distribution of first languages was as follows: The main groups were Bosnian/Croatian/Serbian (23.9%), Albanian (14.1%), Turkish (13.2%), Arabic (6.2%), Romanian (5.8%), and Czech (4.5%). This distribution broadly reflects the proportion of the language groups in the Austrian population of pre-schoolers. Between the cohorts, ages varied between 57.1 and 59.1 months, rate of female participants from 34 to 56%, and length of exposure to German in pre-school from 17.6 to 22.9 months. All the differences reached significance levels [age: *F*_(2, 267)_ = 6.684, *p* < 0.001, η^2^ = 0.048; exposure to the German language: *F*_Welch(2, 164.92)_ = 16.91, *p* < 0.001, η^2^ = 0.089; sex: χ^2^_(2)_ = 8.83, *p* < 0.05, Cramers V = 0.181) demonstrating the diversity of the samples. Children of only two out a total of 27 pre-schools were included in two samples.

**Table 1:** Sample description (family and child characteristics).

	**Sample A**	**Sample B**	**Sample C**	**Total**
Survey year	2014	2015	2017	
Number of pre-schools	12	10	7	27
Sample size	62	96	112	270
Age (months) M (SD)^a^	57.1 (3.65)	59.1 (3.74)	58.9 (3.43)	58.5 (3.67)
Female participants %	34%	56%	55%	50%
Length of institutionalized exposure to the German language (months) M (SD)^b^	17.6 (4.51)	22.9 (7.96)	21.1 (5.66)	20.9 (6.65)

a *Tukey Post-hoc test indicates that Sample A is significantly younger than Samples B and C*.

b *Games-Howell Post-hoc test indicates that length of exposure in Sample A is significantly lower than in Samples B and C*.

#### Procedures

As in Study 1, the screening procedures were carried out by clinical linguists from the Institute for Neurology of Senses and Language and by trained students of speech-language therapy from the University for Health Professions (Fachhochschule für Gesundheitsberufe) in Linz. After the direct assessment of a child, the results were reported to the parent and the pre-school teachers. Within a maximum of 90 days, the children were tested again using standardized reference tests. The tests were again administered by language experts from the Institute of Neurology of Senses and Language who were blinded to the screening results.

#### Measures

##### Screening Measure

The same two screening subscales (EG and RG) were used in Study 2.

##### Reference Tests

In Study 2, we again used the AWST-R to assess expressive vocabulary, and we also used the LiSe-DaZ, a standardized test for assessing German EG and RG with norms for learners of German as L2 (3–7.11 years), accounting for time of German language exposure. In a systematic review of a variety of pre-school language screening instruments and tests in German, the LiSe-DaZ stood out from the other measures by its good differentiation of tasks and its orientation to a model of language acquisition. In the overall evaluation, the test achieved a “very good” result (46). Following Hamann and Abed Ibrahim (27), the classification of LD was used for children who scored a *t*-value of below 38 (i.e., the 10th percentile) in at least two out of nine subtests and below the 10% percentile in the AWST-R (expressive vocabulary test). Based on this classification, 6.7% (*n* = 18) of the children are regarded as having an LD. Supporting the validity of the LD-classification, there is a strong correlation (Phi = 0.538, *p* < 0.001) between LD and a clinical assessment (LD yes/no) made by clinical linguists for the 2017 sample.This assessment was made directly after the administraton of the reference tests including observations of spontaneous language production and interaction, but before scoring. This information is only available for sample C).

##### Feasibility

A short questionnaire (10 items) was developed for screeners to assess time economy, acceptance by children and staff, and practicability in the pre-school setting.

#### Statistical Analyses

We used the same statistical analyses as in Study 2, with two extensions. First, we used confirmatory factor analysis (CFA) for binary items to evaluate the construct validity of the screening scales. The CFA was conducted using weighted least squares estimation (WLSMV) in Mplus 8 (47). Model fit was evaluated following the guidelines proposed by Schermelleh-Engel et al. (48). A good fit is indicated by χ^2^/df ≤ 2, CFI ≥ 0.97, RMSEA ≤ 0.05, and the left boundary of the 90% CI of the RMSEA equals 0. An acceptable fit is indicated by χ^2^/df ≤ 3, CFI ≥ 0.95, RMSEA ≤ 0.08, and a 90% CI close to the RMSEA. As SRMR has been shown to over-reject models for binary indicators (49), we do not report this fit index. Second, we also conducted tests for unpaired ROC curves. We compared ROC curves between subsamples (age groups, sex and length of exposure to the German language). Significant differences between subsamples indicate variations in diagnostic accuracy and limit the generalizability of the screening results (50). In short, we used a bootstrapped test for unpaired ROC curves to compare the AUC of groups for age, sex, and length of exposure to the German language. Additionally, we applied the Venkatraman permutation test (51) that, instead of AUCs, compares actual ROC curves. Notably, if two ROC curves do not differ significantly, cutoff values would result in the same sensitivity and specificity for the subsamples, indicating that a single cutoff would be appropriate for both subsamples.

### Results

#### Descriptive Statistics

**Figure 2:**
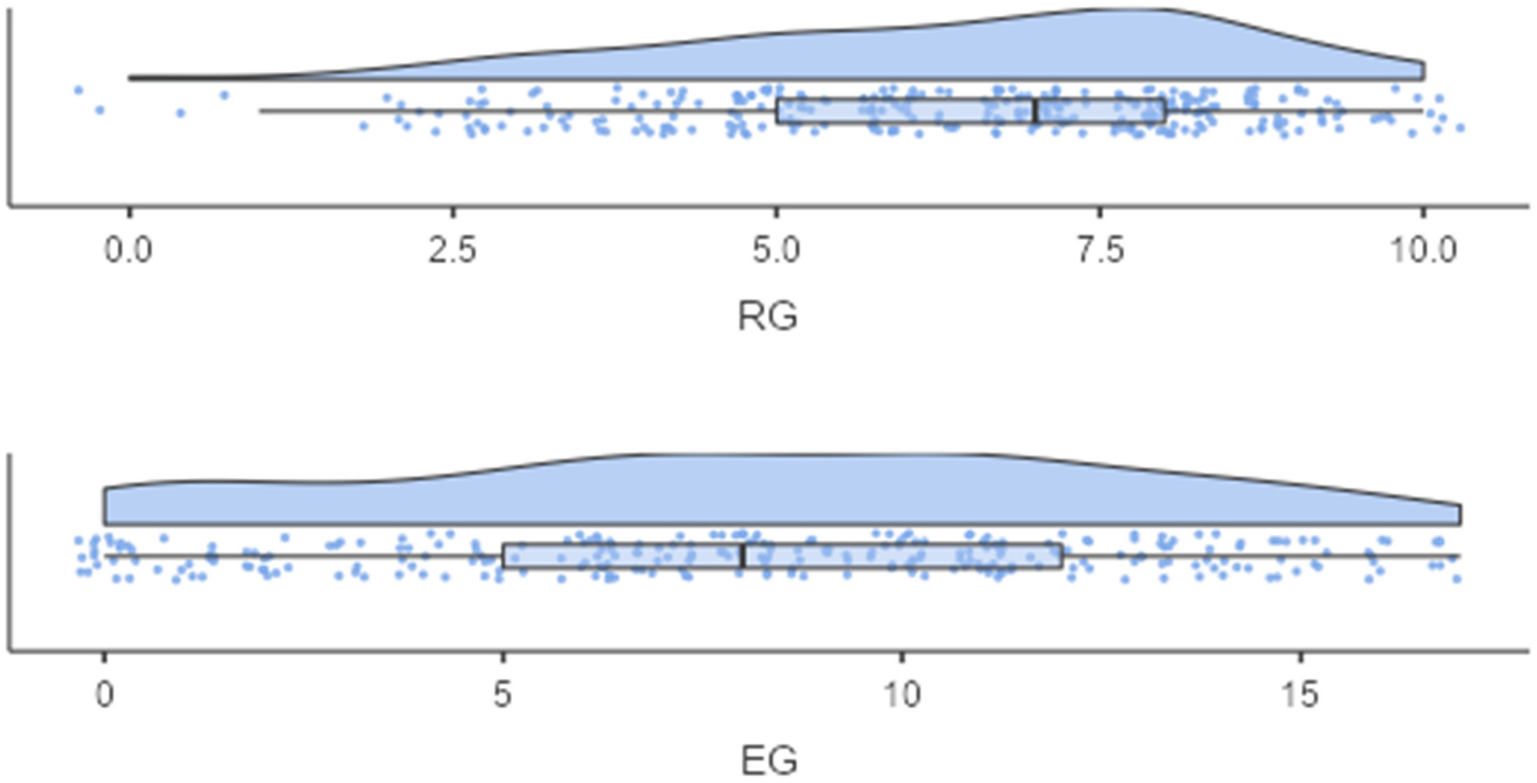
Distribution of the screening subscales—Study 2.

Figure 2 shows the distribution of the screening subscales. Similar to Study 1, the mean of RG (M = 6.38, SD = 2.13) is above the theoretical scale mean of 5. The EG mean (M = 7.86, SD = 4.86) is near the theoretical scale mean of 7.5. However, the EG subscale is again left-censored, indicating that children with low proficiency in EG accumulate at the lower end of the scale. The correlations of screening variables and reference tests are provided as supplement.

#### Reliability

Again, similar to study 1, EG showed good reliability (Cronbach's α = 0.88), and RG repeatedly turned out to have low internal consistency (Cronbach's α = 0.63).

#### Construct Validity

First, we estimated separate single-factor models for RG (M0a) and EG (M0b). Second, we tested a two-dimensional model (RG and EG, M1) against a unidimensional model, where all items load on a single latent variable (i.e., general grammar). Table 2 shows fit indices for the estimated models. The results indicate an acceptable fit for models M0a and M0b. The highly significant (*p* < 0.001) standardized loadings range from 0.34 to 0.79 (median loading = 0.53) for RG and from 0.55 to 0.87 for EG (median loading = 0.70). Furthermore, M1 shows a better fit than M2, supporting the assumption that RG and EG are distinct but highly correlated (latent correlation = 0.87, *p* < 0.001) latent variables.

**Table 2:** CFA-model fit.

	**χ^2^ (*df*)**	**Δχ^2^**	**RMSEA**	**CFI**
M0a: receptive grammar RG (10 Items)	57.052 (35), *p* < 0.05		0.047 (0.023; 0.068)	0.925
M0b: expressive grammar EG (17 Items)	349.575 (119), *p* < 0.001		0.083 (0.073; 0.093)	0.926
M1: 2 Factors (RG and EG)	513.911 (323), *p* < 0.001	–	0.045 (0.038, 0.053)	0.950
M2: 1 Factor (27 Items)	524.707 (324), *p* < 0.001	8.404 (1), *p* < 0.01	0.046 (0.039; 0.054)	0.947

#### Criterion Validity

Table 3 shows the means for children with and without LDs on the screening subscales. In addition, the r_pb_ and AUC are reported. As in Study 1, EG shows an excellent AUC of 0.953 [DeLong 95% CI = (0.904, 1.000)], whereas the AUC for RG is good (0.814). A bootstrapped test for paired ROC curves shows that EG outperforms RG (D = −2.523, *p* < 0.05).

**Table 3:** Descriptive statistics and AUC for the subtests.

	**LD (*n* = 18)**	**No LD (*n* = 252)**	***r*_pb_**	**AUC**	**95% CI (DeLong)**	**Comparison^**a**^**
(1) Screening RG	2.585 (6.482)	6.679 (1.913)	−0.353***	0.814	[0.689–0.940]	
(2) Screening EG	0.667 (1.878)	8.639 (4.411)	−0.423***	0.953	[0.904–1.000]	D = −2.523, *p* = 0.012

*Comparison is based on a bootstrapped test for unpaired ROC curves. r_pb_, point-biserial correlation*.

a Comparison is based on a bootstrapped test for unpaired ROC curves. ***p < .001.

A logistic regression shows that—as in Study 1—only EG significantly predicts LD [b = −0.867, *p* < 0.001; OR = 0.420, 95% CI = (0.267, 0.662)]. The additional effect of RG is insignificant [b = −0.036, *p* > 0.05; OR = 0.964, 95% CI = (0.710, 1.309)], indicating that RG does not have an incremental utility in the prediction of LD. Therefore, the EG subscale seems sufficient as a screening tool.

In the next step, we compared AUC and ROC curves between age groups, sex, and groups defined by the length of institutionalized exposure. Table 4 shows the results. Most notably, AUCs are excellent for all subsamples (>0.90), and we found no significant difference between subsamples. Therefore, these results highlight the generalizability of the diagnostic accuracy across groups and indicate that there is no need for group-specific cutoff values.

**Table 4:** Tests for unpaired ROC curves.

	**AUC**	**95%-CI (DeLong)**	**Comparisons^**a**^**
**A – comparing age groups (median split)**		
(1) <59 months	0.968	[0.943–0.994]	
(2) ≥59 months	0.954	[0.886–1.000]	E = 0.004, *p* = 0.875/D = 0.406, p = 0.685
**B – comparing sex**		
(1) boys	0.980	[0.960–0.999]	
(2) girls	0.918	[0.805, 1.000]	E = 0.008, *p* = 0.140/D = 1.115, *p* = 0.265
**C – comparing LoiE-groups**		
(1) Screening Total (12–18 months inst. German language contact)	0.959	[0.931–0.987]	
(2) Screening Total (19+ month inst. German language contact)	0.944	[0.843–1.000]	E = 0.005, *p* = 0.261/D = 0.296, *p* = 0.767

a *The first test statistic E refers to the Venkatraman test for paired ROC curves. The second test statistic D refers to a bootstrapped test for paired ROC curves. LoiE, Length of individual exposure to German*.

#### Cutoff Estimation

Finally, we again used the “SpEqualSe” criterion (i.e., specificity equals sensitivity) in the Optimal Cutoff Package (40) to determine an optimal cutoff value. The results show that a cutoff value of 1 is the most efficient. This cutoff results in good diagnostic accuracy statistics. Sensitivity and specificity are high at 0.940 [95% CI = (0.727, 0.999)] and 0.936 [95% CI = (0.898, 0.963)], respectively. PPV is 0.515 [95% CI = (0.390, 0.978)], NPV is 0.996 [95% CI = (0.973, 0.998)]. DLR+ and DLR– indicate high confidence in ruling in and ruling out, respectively, a LD. DLR+ is 14.698 [95% CI = (9.031, 23.921)] and DLR– is 0.059 [95% CI = (0.009, 0.399)].

#### Feasibility

The feasibility questionnaire was completed by 42 out of 46 participating speech-language therapists (91.3%) who administered the new screening measure. The assessment of practicabiltiy and acceptance of the screening measure did not differentiate between the new instrument for multilingual children and a version for monolingual German children that had been implemented before, as both versions of LOGiK-S are very similar (materials, procedures). Only administration time was collected specifically for the screening of children with German as their second language. Screening time included the whole procedure including expressive and RG and an additional phonology scale. The results of the phonology scale were not used to contribute to the decision of LD or typical development. Speech-language therapists reported an average screening time of 11.9 min (SD = 4.39; range from 5 to 20 min), demonstrating excellent time economy. The feasibility of LOGiK-S within a regular pre-school setting was considered very good and good by almost all the speech-language therapists, and the efficiency of the new measures (again referring to the comprehensive screening) was assessed as good. High rates of child cooperation and rare child refusal (3%) demonstrated high acceptance of the screening tool by the pre-schoolers. In short, the time economy of the screening and its feasibility in pre-school was assessed as “very good.” According to the operators, the material is designed in an appealing way and was well-accepted by the children. The time required was also rated as satisfactory, as were the personal effort and the personal burden. Around 92% of the participants would recommend the screening to others.

## Discussion

This study investigated the accuracy and feasibility of the newly developed screening measure LOGiK-S in identifying an increased risk of LDs in three sequentially recruited cohorts of bilingual pre-schoolers (*n* = 270, mean age 58.6 months) with German as their second language. A study to develop the screening measures, including initial validation, preceded the comprehensive validation study. The screening was intended for use within the established universal language screening procedure by speech-language-therapists in the penultimate year of pre-school (age 4–5 years) within the regular pre-school settings, and within a constrained time-frame.

The whole study sample was screened and subsequently assessed using standardized language tests. For the validation sample the results of ROC analyses demonstrated high accuracy of the EG screening, with an excellent AUC (0.953). Using a cutoff of 1, the rate of screening fails was 17%, and sensitivity (0.940) and specificity (0.936) were found to be high. In 51.5% (positive predictive value) of these children, a LD was confirmed by standardized language assessments and the application of bilingual norms.

The RG component of the screening did not increase the screening accuracy achieved by the expressive subtest and was therefore regarded as a non-essential component of the screening procedure. However, since limited receptive skills have been found to predict the persistence of LDs (52), the use of receptive screening as a second-step measure for those who screened positive in the EG component might be considered as a tool that helps to better estimate the probability of a persisting LD requiring speech-language therapy. However, for an evidence-based recommendation of a two-step screening, the prospective predictive quality of the receptive measure requires confirmation.

Despite some diversity in the characteristics of the three cohorts (length of L2 exposure, age, and sex) and in pre-school settings (urbanization level), LOGiK-S demonstrated high predictive accuracy in all samples. This can certainly be considered a strength in an instrument to be used with a variety of children in diverse pre-schools. The non-significant effect of length of time of L2 exposure on the screening results may initially be surprising. However, because in many pre-schools attended by children with German as an L2, a high number, and often the majority of their peers, have family languages other than German, it is very likely that—despite pre-school attendance—the daily quantity of high-quality German language input and particularly the amount of active participation in language interactions in German is limited and highly variable. The quality and quantity of everyday L2 input in the pre-school from peers and caregivers can most likely be considered more relevant to L2 development than the length of L2 exposure (53–55).

Although ASHA (56) proposes that bilingual children be assessed in both languages, a number of practical constraints render the implementation of the guidelines difficult or even impossible. Even obtaining reliable information on first language acquisition and L2 language exposure of all pre-school children with German as a second language is hardly feasible. The present results show that testing in the majority language with norms for learners as L2 can be regarded as a practical and accurate alternative.

## Limitations

The high number of children attending a pre-school with an accumulation of learners of German as an L2 might be considered a limitation of this study because our findings might not be generalizable to the total population of children with a L1 other than German. On the other hand, the majority of children with German L2 acquisition in Austria representing the target group for the screening live in urbanized areas and attend pre-schools with a high percentage of children with migrant backgrounds. The exclusion of children attending the first year of pre-school is a limitation. However, our results show that despite their exclusion, simple EG items were challenging for many bilingual children, as demonstrated by the low cutoff. The lack of a well-defined gold standard for LDs in general and—more specifically—in bilingual children must still be regarded as a significant challenge for developing screening measures.

## Conclusion

The LOGiK-S EG screening is feasible and identifies LD in children with a variety of first languages other than German. Using a screening measure focusing on the acquisition of German expressive grammar applying specific bilingual norms allows for reliable differentiation between children with and without LDs, even though standardized first language testing is not practical.

## Author's Note

DH is a clinical linguist and director of the center for communication and language at the Institute of Neurology of Senses and Language at the Hospital of St. John of God. His research interests concern the early identification of developmental disorders, the efficacy of interventions in disorders of speech/language/communication, and the association between communication skills and mental health. CW is a social scientist and is working at the University of Education Upper Austria and the Research Institute for Developmental Medicine, Johannes Kepler University Linz. His research interests are quantitative methods and educational inequalities. MJ is a clinical linguist working at the Institute for Neurology of Senses and Language at the Hospital of St. John of God in Linz (Austria). She specializes in the field of diagnosing speech, language, and communication needs in monolingual and bilingual children.

## Data Availability Statement

The datasets presented in this article are not readily available because parents have not given their consent to data sharing. Requests to access the datasets should be directed to daniel.holzinger@bblinz.at.

## Ethics Statement

The studies involving human participants were reviewed and approved by Ethikkommission Barmherzige Schwestern und Barmherzige Brüder, Linz. Written informed consent to participate in this study was provided by the participants' legal guardian/next of kin.

## Author Contributions

DH: conceptualization and formal analysis. CW and DH: methodology. MJ: data curation. MJ, DH, and CW: writing original draft and review and editing. DH and MJ: project administration. All authors contributed to the article and approved the submitted version.

## Funding

This work was supported by the Department of Social Affairs of the Upper Austrian Government. Article processing charge is funded by the Johannes Kepler University Open Access Publishing Fund.

## Conflict of Interest

The authors declare that the research was conducted in the absence of any commercial or financial relationships that could be construed as a potential conflict of interest.

## Publisher's Note

All claims expressed in this article are solely those of the authors and do not necessarily represent those of their affiliated organizations, or those of the publisher, the editors and the reviewers. Any product that may be evaluated in this article, or claim that may be made by its manufacturer, is not guaranteed or endorsed by the publisher.
